# Protocol for co-producing a framework and integrated resource platform for engaging patients in laboratory-based research

**DOI:** 10.1186/s40900-024-00545-7

**Published:** 2024-02-12

**Authors:** Manoj M. Lalu, Dawn Richards, Madison Foster, Brittany French, Angela M. Crawley, Kirsten M. Fiest, Kathryn Hendrick, Kimberly F. Macala, Asher A. Mendelson, Pat Messner, Stuart G. Nicholls, Justin Presseau, Cheryle A. Séguin, Patrick Sullivan, Bernard Thébaud, Dean A. Fergusson

**Affiliations:** 1 Blueprint Translational Research Group, Clinical Epidemiology Program, The Ottawa Hospital Research Institute, Ottawa, ON Canada; 2 Regenerative Medicine Program, The Ottawa Hospital Research Institute, Ottawa, ON Canada; 3 Department of Anesthesiology and Pain Medicine, The Ottawa Hospital, Ottawa, ON Canada; 4 Five 02 Labs Inc, Toronto, ON Canada; 5 Patient Partner, Toronto, ON Canada; 6 Chronic Disease Program, The Ottawa Hospital Research Institute, Ottawa, ON Canada; 7 Department of Biochemistry, Microbiology and Immunology, University of Ottawa, Ottawa, ON Canada; 8 Centre for Infection, Immunity and Inflammation, University of Ottawa, Ottawa, ON Canada; 9 Department of Critical Care Medicine, University of Calgary, Calgary, AB Canada; 10 Departments of Critical Care Medicine and Anesthesiology and Pain Medicine, Royal Alexandra Hospital, Edmonton, AB Canada; 11 Section of Critical Care Medicine, Department of Medicine, Rady Faculty of Health Sciences, University of Manitoba, Winnipeg, MB Canada; 12 Patient Partner, Ottawa, ON Canada; 13 Ottawa Methods Centre, The Ottawa Hospital Research Institute, Ottawa, ON Canada; 14 School of Epidemiology and Public Health, University of Ottawa, Ottawa, ON Canada; 15 Department of Physiology and Pharmacology, Schulich School of Medicine and Dentistry, The University of Western, London, ON Canada; 16 Patient Partner, Vancouver, BC Canada; 17 Department of Cellular and Molecular Medicine, University of Ottawa, Ottawa, ON Canada; 18 Clinical Epidemiology and Regenerative Medicine Programs, BLUEPRINT Translational Research Group, The Ottawa Hospital Research Institute, Room B307, 1053 Carling Ave, Mail Stop 249, Ottawa, K1Y 4E9 Canada; 19 Department of Medicine, University of Ottawa, Ottawa, Canada; 20 Department of Pediatrics, Children’s Hospital of Eastern Ontario (CHEO) and CHEO Research Institute, Ottawa, Canada

**Keywords:** Patient engagement, Preclinical research, Basic science, Laboratory research, Preclinical laboratory research, Patient and public involvement, Patient oriented research, Deliberative knowledge space, Delphi survey, Pilot field test, Framework, Consumer involvement

## Abstract

**Background:**

Patient engagement in research is the meaningful and collaborative interaction between patients and researchers throughout the research process. Patient engagement can help to ensure patient-oriented values and perspectives are incorporated into the development, conduct, and dissemination of research. While patient engagement is increasingly prevalent in clinical research, it remains relatively unrealized in preclinical laboratory research. This may reflect the nature of preclinical research, in which routine interactions or engagement with patients may be less common. Our team of patient partners and researchers has previously identified few published examples of patient engagement in preclinical laboratory research, as well as a paucity of guidance on this topic. Here we propose the development of a process framework to facilitate patient engagement in preclinical laboratory research.

**Methods:**

Our team, inclusive of researchers and patient partners, will develop a comprehensive, empirically-derived, and stakeholder-informed process framework for ‘patient engagement in preclinical laboratory research.’ First, our team will create a ‘deliberative knowledge space’ to conduct semi-structured discussions that will inform a draft framework for preclinical patient engagement. Over the course of several sessions, we will identify actions, activities, barriers, and enablers (e.g. considerations and motivations for patient engagement in preclinical laboratory research, define roles of key players). The resulting draft process framework will be further populated with examples and refined through an international consensus-building Delphi survey with patients, researchers, and other collaborator organizations. We will then conduct pilot field tests to evaluate the framework with preclinical laboratory research groups paired with patient partners. These results will be used to create a refined framework enriched with real-world examples and considerations. All resources developed will be made available through an online repository.

**Discussion:**

Our proposed process framework will provide guidance, best practices, and standardized procedures to promote patient engagement in preclinical laboratory research. Supporting and facilitating patient engagement in this setting presents an exciting new opportunity to help realize the important impact that patients can make.

**Supplementary Information:**

The online version contains supplementary material available at 10.1186/s40900-024-00545-7.

## Abstract (plain language summary)

Engaging patients as partners or collaborators in clinical research is becoming more common, but it is still new in preclinical research. Preclinical researchers work in laboratories on cell and animal experiments. They traditionally don’t have frequent interactions with patients compared to their clinical research colleagues. Integrating patient engagement in preclinical laboratory research may help ensure that patient perspectives and values are considered. To help preclinical laboratory research align with patient-centred priorities we propose the development of a practical framework. This framework will facilitate patient engagement in preclinical laboratory research. To achieve this, we will first hold in-depth discussions with patient partners, researchers, and other collaborators to understand views on patient engagement in preclinical laboratory research. Together, we will identify key considerations to draft a framework, including motivations for patient engagement in preclinical laboratory research, and defining the roles of those who need to be involved. We will refine the framework through an international survey where we will collect feedback from researchers, patient partners, and other collaborators to make further improvements. The framework will then be tested and refined by preclinical laboratory teams inclusive of patient partners. The finalized framework and other resources to facilitate patient engagement in preclinical laboratory research will be hosted in a ‘one-stop-shop’ of online resources. Ultimately, this framework will enable partnerships between patients and researchers and provide a roadmap for patient engagement in preclinical laboratory research. This presents an exciting new opportunity for patients and researchers to collaborate and potentially improve translation of laboratory-based research.

**Supplementary Information:**

The online version contains supplementary material available at 10.1186/s40900-024-00545-7.

## Introduction

Although patient engagement has been adopted and recognized for its benefits in clinical research, little is known about its prevalence and effects in ‘preclinical’ research [1, 2]. This type of research, performed in laboratories using cells, tissues, or animals, informs decisions to move treatments into clinical trials. Engaging patient partners (i.e., individuals with lived experience of a health condition, including informal caregivers, family, and friends [3]) could ensure preclinical laboratory research aligns with patient preferences and priorities. Indeed, the primary motivators underlying preclinical research are the health conditions experienced by patients. Despite these potential benefits, there is a paucity of available frameworks, resources, or guidance documents available to researchers or patient partners in regard to patient engagement in preclinical laboratory research [1, 2].

To begin to address this knowledge gap, our team previously conducted a scoping review and interview study to map and understand current patient engagement practices in preclinical laboratory research [1, 4]. We identified 32 reports, which demonstrated that engagement in the laboratory setting was feasible. In addition, meaningful benefits similar to those seen in patient engagement in clinical research were identified, such as the exchange of diverse perspectives and creation of bi-directional learning opportunities [4]. Preclinical researchers were given the opportunity to learn about lived experiences for the health conditions they study and how therapies impact patients. It was also suggested that engagement could enhance preclinical researcher communication and motivation for their work [1, 4, 5]. In turn, patients were able to learn more about their health condition, ongoing projects, and challenges to research in the field [1, 4].

We also identified barriers and challenges to laboratory-based preclinical patient engagement. For instance, preclinical research is not typically public-facing, and preclinical laboratory researchers do not routinely interact with patients as part of their regular research duties [1, 4]. As a result, the benefits of patient engagement in preclinical laboratory research may be less intuitive compared to clinical research. In addition, communication between preclinical researchers and patients can also be challenging as it requires use of a shared vocabulary and identification of common goals [1, 4].

Due to the novelty and challenges of patient engagement in preclinical laboratory research, the question of *how* and *when* patients can be most meaningfully engaged in the process remains unclear. Our team of preclinical and clinical researchers and patient partners propose to co-create, refine, and pilot test a framework to address these issues [7]. Drawing upon insights from implementation science [7], we aim to co-create a *process* framework, which will provide key steps to implementing patient engagement in preclinical laboratory research. Our framework will synthesize collaborator views, including input from an international advisory board, with our findings from previous work [1, 4] and offer practical guidance for planning and execution of ‘laboratory-based preclinical patient engagement.’

**Fig. 1:**
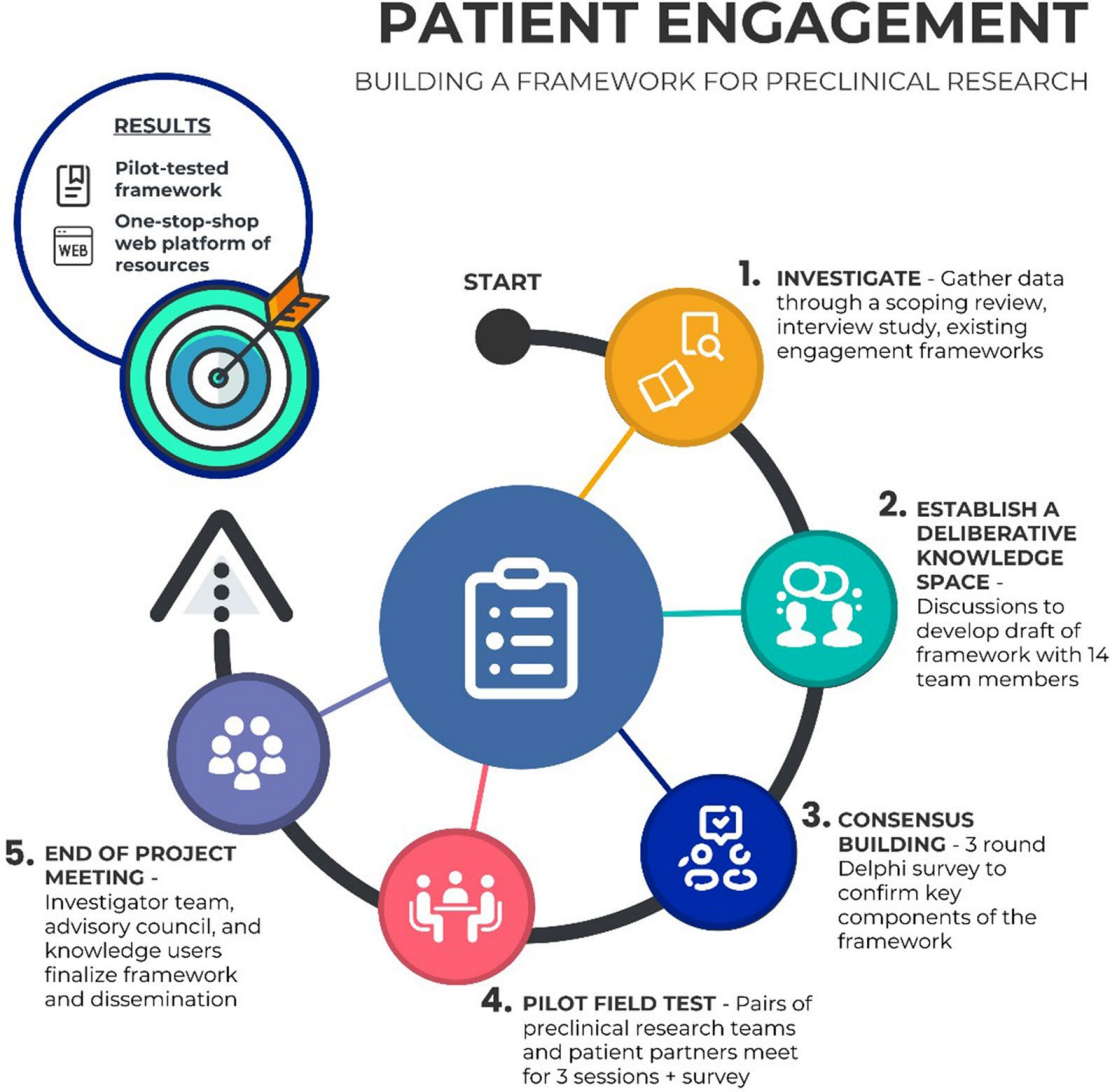
Proposed steps to create a process framework for patient engagement in laboratory-based preclinical research

Here we describe a protocol for a series of studies to develop and test our framework (see Fig. 1). First, we will conduct a series of ‘deliberative knowledge space’ meetings. These will be facilitated discussions with patient partners, preclinical researchers, and experts in patient engagement to exchange ideas on creating our draft framework. Results from the deliberative knowledge space meetings and the initial draft framework will also be shared with an international advisory board, to obtain additional input and feedback. We will next conduct a modified multi-round Delphi survey with international collaborators to further refine the draft framework. The final round will constitute a facilitated, virtual ‘face-to-face’ meeting, which is a common Delphi modification thought to promote discussion and clarity [8, 9]. Finally, we will evaluate the framework through pilot field testing with collaborating patient partners and laboratory-based preclinical research teams. This work will lead to a user-informed generalizable framework. We will disseminate and implement this framework by working with international collaborators and creating an online repository of resources. Our proposed preclinical patient engagement framework will promote the involvement of patients at the earliest stages of the research continuum.

## Methods/Design

### Development of the initial draft process framework

As noted, process frameworks provide guidance on the implementation of knowledge into action, breaking the practice down into stages and assigning actions at each stage [7]. Consequently, our initial step will be to map out the key stages in the preclinical laboratory research process—from project conception and planning, through to implementation and reporting—and identify potential areas for patient engagement. As a starting point, we will work from (a) several existing clinical frameworks identified by members of our institutional patient engagement support unit, (b) the evidence from our scoping review and qualitative interview studies [1, 4], as well as (c) patient partner and collaborator input to date. To draw upon diverse perspectives and experiences, we have assembled a multidisciplinary team of fourteen investigators, including patient partners with lived experience of a condition or caregiving, researchers with expertise in preclinical laboratory research, clinical research, or qualitative research, and a patient engagement facilitator. Using the noted sources our team will co-develop a draft process framework that outlines at what stages and to what capacity patient engagement could be introduced to preclinical laboratory research.

### Patient engagement within the team

Our team encompasses a patient Co-Principal Investigator (DR) and three patient Co-Investigators (KH, PM, PS). Over the last four years, DR, KH, PM, and PS have been members of the team working on various components of the research program including developing, writing and editing five successful grant applications for the program, improving the scoping review protocol, interview guide, and recruitment documents. They offer valuable insights on feasibility of study procedures and delivery of informational resources; they have also helped clarify *why* and *how* patients can be engaged at this early stage of research. In the current project, patient partners will be involved throughout all study aspects, such as protocol development, team meetings, framework development, authorship of study materials and questionnaires, recruitment strategies, framework refinement, pilot testing sessions, and manuscript development, depending on individual interest and availability. Patient partners will be recognized for contributions based on guidance from the SPOR Evidence Alliance’s Patient Partner Appreciation Policy and Protocol [10] and remunerated according to Canadian Institutes of Health Research guidelines [11]).

Recognizing the importance of patient partner compensation, all patient participants who contribute to the Delphi survey and pilot-field tests will be compensated and recognized in alignment with best practices [10–12]. Researcher and ‘other’ participants will be offered a gift card as a token of appreciation.

### Deliberative knowledge space meetings

To systematically draw from these sources and build consensus on what should be included within the draft framework, our team of researchers and patient partners will begin with a series of ‘deliberative knowledge space’ meetings. Deliberative knowledge spaces have been defined as a metaphorical space that provides participants with the resources to consider and discuss an issue in depth in order to develop a considered view [13]. This approach has been used by Staniszewska and colleagues to co-develop a framework for public engagement in economic modelling, which is a similarly largely non-public-facing field of research [14]. We will follow INVOLVE’s nine principles for deliberative engagement (see Additional file 1) [13]. Though there is no one specific approach, the INVOLVE principles and Staniszewska et al. suggest tailoring the structure to the objectives, designating adequate time for collaborator discussion [13], and providing adequate information on the topic so that collaborators are encouraged to share informed perspectives and ask questions [14].

Following this guidance, we have designed four deliberative knowledge space meetings that will be held virtually through videoconferencing. We will initiate semi-structured discussion by providing background information on patient engagement and preclinical laboratory research. This will be followed by question prompts that facilitate conversation, followed by breakout room discussion among participants. Discussion points during the meetings will include: identifying motivations for engagement, possible roles and actions/activities, expectations and potential outcomes for both patient partners and preclinical researchers, and barriers and enablers to patient engagement in preclinical laboratory research. We will also consider process issues that should be included in the framework, such as recruitment of patient partners, identification of key actors, and outlining the stages of preclinical laboratory research.

Discussions will be documented through recordings, meeting minutes, and narrative summaries [14]. Underlying guiding key themes and principles will be synthesized and used to inform a working draft of the process framework, which will also be supplemented with case studies, examples, and resources. The working draft will then be refined through offline communication and further meetings, as needed. Results from the deliberative knowledge space meetings and the initial draft framework will also be shared with an International Advisory Board, which consists of an international patient partner and four patient engagement researchers, to obtain additional perspectives and input.

### Consensus building Delphi survey

To refine the framework, we will collect and integrate input from a wide range of international collaborators using a modified three-round, remote Delphi survey. This method was chosen due to the geographical spread of potential participants, as well as the use of anonymous voting that minimizes group power dynamics. Through the Delphi survey, identified international collaborators with an interest or expertise in preclinical patient engagement will be asked to identify and rate the importance of specific items identified by our draft framework (e.g., an effective way of building relationships, creating a common vocabulary, considering patients’ involvement throughout the research cycle). Consensus on items determined to be important or unimportant will allow our team to focus our efforts on elaborating and expanding the framework in collaborator-identified priority areas. For instance, some collaborators have indicated the potential for patient engagement to help better communicate how and why animals may be used in biomedical research.

### Participants and recruitment

The Delphi survey will span three broad respondent groups: patient partners (inclusive of patients and their friends and family caregivers), researchers (inclusive of investigators, trainees, and highly qualified personnel), and ‘others’ (funders, network leads, animal care committees, etc.). We will aim for each group to have a minimum of 10 participants, in line with the suggested range [15]. Purposive sampling will be used. Potential participants will be identified through collaborating organizations with an interest in applying findings from this work and who have committed to providing support throughout the project. These groups encompass patient organizations and panels (e.g. Canadian Cancer Stakeholder Alliance), funders (e.g. the United Kingdom’s National Institute for Health and Care Research), research networks (e.g. Stem Cell Network, BioCanRx), and charities (e.g. Versus Arthritis) from multiple countries. Additional participants will also be identified through our scoping review [1] (representing teams from the United Kingdom, the Netherlands, United States, Germany, Italy, Ireland and Canada), interview studies [4], our International Advisory Board Members (representing teams from the United Kingdom, Ireland and Canada), social media as well as snowball sampling through collaborators and survey participants. Individuals with prior experience of (a) at least one preclinical laboratory patient engagement initiative, or (b) experience with more than one patient engagement in health research initiative, or (c) a background in preclinical laboratory research (undergraduate degree or higher) will be eligible to participate.

### Development of the survey

Our team of researchers and patient partners will draw upon existing literature and our previously conducted research to generate items for the initial survey. Components of the draft framework (e.g. important considerations, specific activities) will be operationalized into items. A draft version will then be shared with designated team members for preliminary feedback and item reduction/refinement. We also plan to pilot the survey with advisory board members to assess for face validity.

### Conduct and analysis

#### Round one

All three rounds will be conducted using the cloud-based Delphi software SurveyLet® [Calibrum, St. George, Utah]. The online survey will begin with a short video that summarizes key concepts, the draft framework, and optional resources. This short video was suggested by team patient partners and will be co-developed with them. Participants will anonymously rate items on a 9-point scale (7–9 points = essential to include within the framework, 4–6 = potentially essential, and 1–3 points = unnecessary). Survey participants may enter free text to provide rationale, comments, or propose additional items. Suggestions for new items will be included in subsequent rounds of the survey, while aggregated comments will be reported alongside the associated item for participants to view. The research team will analyze the results to determine whether each item has reached the threshold for consensus. Thresholds for consensus will be greater than 80% of respondents scoring 7–9, indicated as important to include, or 1–3, indicated as not important and should be excluded [16].

#### Round two

A new iteration of the survey will be developed. Items that did not meet the threshold for consensus will be included. For each item, participants will be provided their individual rating from the first round as well as the overall group’s median rating. They will then be asked to re-rate the item. Any newly identified items from Round One will also be included.

#### Round three (virtual face-to-face)

Our revised Delphi process will culminate in a third and final round, which will be conducted virtually through a facilitated face-to-face voting session [8]. This is a common modification to promote discussion and clarification [9]. Participants will meet virtually to discuss and clarify any questions surrounding the remaining items. The discussion will be co-facilitated by a member of our research team and a patient partner. This session will also include multiple breakout sessions to allow for discussion. The meeting will be recorded and transcribed. Voting will then take place during the meeting and remain anonymous using real-time features in SurveyLet®.

The response rate will be documented for each round. Answers to free text responses will be analyzed by two reviewers to assess whether a new item has been suggested. Secondary exploratory analysis of the results across demographic data will be performed on factors such as gender, location, respondent group(s) represented, stage of career for researchers, and number of experiences with patient engagement. Final results from the Delphi survey will be used to update and refine the framework.

### Pilot field testing

To facilitate its implementation, we will evaluate our framework through pilot field testing with ten groups of preclinical laboratory researchers paired with patient partners. Pilot field testing will identify barriers and facilitators to this process, and highlight opportunities that should be explored further to refine the framework.

### Participants and recruitment

Ten preclinical laboratory research teams and twenty patient partners will be recruited. Each of the ten groups will include the principal investigator of the research team and one to two team members (e.g. research associate, trainee), matched to two patient partners. We have selected this number of participants for feasibility, balance between researcher and patient partner team members, and the aim of fostering meaningful partnerships between team members. No prior experience with preclinical laboratory research (for patient partners) or patient engagement (researchers or patient partners) will be required. Researchers with either an active or upcoming project will be identified through one of our funders (Stem Cell Network) and professional networks. We will assist with identification of patient partners with lived experience matched to the research teams through collaborator organizations (e.g. Cancer Stakeholder Alliance, Sepsis Canada). We will also organize meetings and onboarding with patient partners. As we anticipate a learning curve for implementation of the framework, pilot field testing will be initiated sequentially in order to incorporate feedback in an iterative manner.

### Educational sessions and applying the framework (introduction, discussion, implementation and planning)

Pilot field testing of the framework for each group will be initiated over three consecutive sessions (in-person if possible, or virtually). We anticipate the process described will be refined based on the findings of the deliberative knowledge space and Delphi survey. Each session will be facilitated by a research assistant trained in patient engagement as well as our draft process framework. In Session #1- ‘Introduction,’ the preclinical researchers and patient partners will be provided background information on patient engagement and preclinical laboratory research. In addition, the process framework will be introduced. Next, researchers will have an opportunity to describe their research, followed by a chance for patient partners to share aspects of their lived experience. A ‘take-home’ exercise will be provided; both researchers and patient partners will be asked to use the framework to generate ideas on how they may work together.

The first part of this session aims to introduce preclinical researchers to key values of patient engagement and facilitate patient partner familiarity and understanding of the preclinical research process, the research continuum, applicable regulatory requirements and other relevant concepts or techniques. To provide more in-depth training, all team members will also be required to complete the Canadian Institutes of Health Research Institute of Musculoskeletal Health and Arthritis Patient Engagement in Research Modules (resources co-developed by co-lead DR, [17]) after the session. This free, online course aims to help patient partners, researchers, trainees, and other team members understand patient engagement in research through four modules: (1) What is patient engagement? (2) The research process: (a) Understanding the research process for patient partners and (b) Supporting patient partners throughout the research process for other members of the team, (3) Setting up a research project for successful partnership and (4) Patient engagement for research teams: (a) Being part of a research team for patient partners and (b) Engaging patients on your research team for other members of the research team.

In Session #2- ‘Discussion of Potential Methods to Work Together,’ participants will be encouraged to refer to the framework and their ‘take-home’ exercise to help guide discussion on opportunities for engagement in the preclinical research project. This session will be semi-structured; we will start with an open discussion to allow participants the opportunity to share their ideas from the ‘take-home’ exercise. Members of our team will then facilitate further discussion, using a co-developed list of prompts, to build on these ideas and incorporate suggested strategies from the framework and identified case studies. The session will then close with any remaining thoughts, questions, or concerns from the researchers and patient partners.

Finally, in Session #3- ‘Implementation and Planning,’ each team will work to co-develop action plans that will outline immediate first steps to initiate identified activities. Through these discussions, each team will also begin to develop a terms of reference [18] and potentially make longer-term plans for their patient engagement strategy. During this time, research assistants will capture and document feedback on the framework, through meeting minutes and impact logs.

### Post-Pilot field testing survey

After the third session, each group member will assess our framework through an online survey based on the Theoretical Framework of Acceptability (TFA) [19]. The TFA is an adaptable tool that seeks to evaluate the acceptability of healthcare interventions across eight domains: (1) Affective Attitude (how an individual feels about an intervention), (2) Burden (the amount of effort required to participate in the intervention), (3) Ethicality (does the intervention align with an individual's values?), (4) Perceived Effectiveness (does the intervention achieve its purpose?), (5) Intervention Coherence (does the individual understand how the intervention works?), (6) Self-Efficacy (is the individual confident they can use the intervention?), (7) Opportunity Costs (the benefits, profits, or value that was given up to engage with the intervention) and (8) General Acceptability [19]. To assess the quality of the engagement partnerships and potential impacts, we will additionally include questions from the Public and Patient Engagement Evaluation Tool [20]. The survey will also collect information on the research area, and researcher/patient partner characteristics. This will allow us to assess, in an exploratory manner, if stage of research, funding, or laboratory size, may influence the *when* and *how* of preclinical patient engagement.

### Survey data analysis

Quantitative survey data will be summarized by descriptive statistics. Open-text survey questions and documented minutes will be analyzed using thematic content analysis [21]. We will analyze differences between groups, researchers, and patient partners. Feedback will be categorized and assessed for inclusion based on whether it could be addressed through revisions in the framework. This will be completed by one research assistant and verified by a patient partner. Key feedback categories will then be arranged into tables so high-level themes can be identified independently by two team members. Established themes will be discussed by our team, and iteratively modified as needed.

### Refining/finalizing the framework

Results will be used to make refinements to the draft framework, to ensure all relevant aspects are included, as well as identify priority areas or knowledge gaps for future research. This refined framework will then be presented to collaborators for further feedback, approval, and dissemination.

### Dissemination and future directions

To ensure wide uptake of our framework, we will employ multiple strategies to disseminate our final products. First, we plan to have regular meetings to share information and ensure maximal engagement amongst all collaborators and contributors. We will publish our work in open access, peer-reviewed journals as manuscripts describing (1) the final version of the framework and its development, and (2) an explanation and elaboration document to provide specific examples and case studies for each component of the framework. Both manuscripts will also be co-produced with patient partner team members. We will use appropriate reporting guidelines for each manuscript to ensure transparency and completeness (e.g. Sex and Gender Equity in Research guidelines [22] and Guidance for Reporting Involvement of Patients and the Public (GRIPP2) [23]).

To reach a wider audience we will also create an online resource for patient engagement in preclinical laboratory research, which will link to the Ottawa Hospital Research Institute’s Office for Patient Engagement in Research Activities (OPERA) website. This will be curated to target three groups: organizations and institutions, preclinical researchers, and patient partners. It will provide access to the framework and other resources and tools developed by our team and other organizations (e.g. videos, infographics, helpful readings, and tools). We will also create a non-technical summary for each study to post on various social media platforms and blogs (e.g. Ontario Strategy for Patient-Oriented Research SUPPORT Unit Blog), as well as our website. These products will be co-developed with patient partners to ensure the use of a common vocabulary.

Importantly, our collaborator organizations have a demonstrated need for products of this project and have indicated that they are eager to help disseminate the resources and find opportunities to implement the framework. Moreover, our research team includes collaborators from five Canadian universities, each of whom have significant involvement in various research societies and are ideally situated to further promote dissemination and uptake. In addition to providing practical guidance on how patients and preclinical researchers can work together, our hope is that our project deliverables will contribute to a broader cultural shift. Through our dissemination strategies, we aim to raise awareness of the importance of involving patients in preclinical laboratory research. Integration of trainees in this process may also effect a meaningful change to training. Future research will then work towards further refinement of the framework, implementation, and assessment of its impacts.

## Discussion

Our proposed approach will systematically account for perspectives from various key collaborators and knowledge users. This series of studies will also provide evidence to inform and refine our process framework. Moreover, through our knowledge translation efforts to disseminate our final developed framework, we will help preclinical researchers and patients to establish relationships and to identify activities, methods, and further considerations for engagement. Promotion of laboratory-based preclinical patient engagement will help align preclinical research priorities to patient needs and experiences, allow patients to have a better understanding of the nuances of this non-public facing domain of science, and expose preclinical laboratory researchers to real-lived experiences of the conditions they study.

### Supplementary Information

**Additional file 1. Table S1**. INVOLE’s Nine Principles for Deliberative Engagement.

## Data Availability

Not applicable. All materials and datasets generated or analyzed during the proposed study will be made available in the subsequent study manuscripts or from the correspondence author on reasonable request. Recordings of deliberative knowledge space meetings and pilot field tests, as well as individual survey responses will be kept private to respect participant privacy.

## Abbreviations

**TFA**: Theoretical Framework of Acceptability
**GRIPP2**: Guidance for Reporting Involvement of Patients and the Public
**OPERA**: Office for Patient Engagement in Research Activities
**PPEET**: Public and Patient Engagement Evaluation Tool

## References

[1] Fox G, Fergusson DA, Daham Z, Youssef M, Foster M, Poole E (2021). Patient engagement in preclinical laboratory research: a scoping review. EBioMedicine.

[2] Maccarthy J, Guerin S, Wilson AG, Dorris ER (2019). Facilitating public and patient involvement in basic and preclinical health research. PLoS ONE.

[3] Government of Canada - Canadian Institutes of Health Research. Patient engagement—CIHR [Internet]. 2012 [cited 2022 Dec 23]. Available from: https://cihr-irsc.gc.ca/e/45851.html.

[4] Foster M, Fergusson DA, Thompson E, Hunniford V, Richards DP, Messner P, et al. Engaging patients in preclinical laboratory research: an interview study. Prep. 2022. 10.1186/s40900-025-00818-9 PMC12723845 41316501

[5] Carroll P, Dervan A, Maher A, McCarthy C, Woods I, Kavanagh R (2022). Applying Patient and Public Involvement in preclinical research: a co-created scoping review. Health Expect Int J Public Particip Health Care Health Policy.

[6] Greenhalgh T, Hinton L, Finlay T, Macfarlane A, Fahy N, Clyde B (2019). Frameworks for supporting patient and public involvement in research: systematic review and co-design pilot. Health Expect.

[7] Nilsen P (2015). Making sense of implementation theories, models and frameworks. Implement Sci.

[8] Boulkedid R, Abdoul H, Loustau M, Sibony O, Alberti C (2011). Using and reporting the Delphi method for selecting healthcare quality indicators: a systematic review. PLoS ONE.

[9] Moher D, Schulz KF, Simera I, Altman DG (2010). Guidance for developers of health research reporting guidelines. PLOS Med.

[10] SPOR Evidence Alliance. Patient Partner Appreciation Policy and Protocol. 2019.

[11] Government of Canada - Canadian Institutes of Health Research. Considerations when paying patient partners in research - CIHR [Internet]. 2019 [cited 2023 Oct 23]. Available from: https://cihr-irsc.gc.ca/e/51466.html.

[12] Fox G, Fergusson DA, Sadeknury A, Nicholls SG, Smith M, Stacey D (2024). What guidance exists to support patient partner compensation practices? A scoping review of available policies and guidelines. Health Expect.

[13] National Consumer Council and INVOLVE (2008). Deliberative public engagement: nine principles.

[14] Staniszewska S, Hill EM, Grant R, Grove P, Porter J, Shiri T (2021). Developing a framework for public involvement in mathematical and economic modelling: bringing new dynamism to vaccination policy recommendations. Patient.

[15] Hsu CC, Sandford BA (2007). The Delphi technique: making sense of consensus. Pract Assess Res Eval.

[16] Diamond IR, Grant RC, Feldman BM, Pencharz PB, Ling SC, Moore AM (2014). Defining consensus: a systematic review recommends methodologic criteria for reporting of Delphi studies. J Clin Epidemiol.

[17] Government of Canada - Canadian Institutes of Health Research. Patient Engagement: Patient Engagement Training—CIHR [Internet]. 2005 [cited 2024 Jan 18]. Available from: https://cihr-irsc.gc.ca/e/27297.html.

[18] National Institute for Health and Care Research (NIHR), INVOLVE. Terms of Reference Template 1 —NIHR [Internet]. 2012 [cited 2024 Feb 7]. Available from: https://www.invo.org.uk/terms-of-reference-template-1-5/.

[19] Sekhon M, Cartwright M, Francis JJ (2022). Development of a theory-informed questionnaire to assess the acceptability of healthcare interventions. BMC Health Serv Res.

[20] Abelson J. The Public and Patient Enagagement Evaluation Tool. 2015.

[21] Braun V, Clarke V (2006). Using thematic analysis in psychology. Qual Res Psychol.

[22] Heidari S, Babor TF, De Castro P, Tort S, Curno M (2016). Sex and Gender Equity in Research: rationale for the SAGER guidelines and recommended use. Res Integr Peer Rev.

[23] Staniszewska S, Brett J, Simera I, Seers K, Mockford C, Goodlad S (2017). GRIPP2 reporting checklists: tools to improve reporting of patient and public involvement in research. BMJ.

