# Supplementary material for: Protocol for co-producing a framework and integrated resource platform for engaging patients in laboratory-based research
Source: Res Involv Engagem. 2024 Feb 12;10:25. doi: 10.1186/s40900-024-00545-7 (PMC10863123; doi:10.1186/s40900-024-00545-7)
Supplement: Supplementary file 1 — Additional file 1. Table S1. INVOLE’s Nine Principles for Deliberative Engagement. [file 40900_2024_545_MOESM1_ESM.docx]

**Supplemental File 1.**

Table 1. INVOLE’s Nine Principles for Deliberative Engagement.

| **No.** | **Principle** |
| --- | --- |
| 1. | The process makes a difference. |
| 2. | The process is transparent. |
| 3. | The process has integrity. |
| 4. | The process is tailored to circumstances. |
| 5. | The process involves the right number and types of people. |
| 6. | The process treats participants with respect. |
| 7. | The process gives priority to participants’ discussions. |
| 8. | The process is reviewed and evaluated to improve practice. |
| 9. | Participants are kept informed. |
